# The value of cystatin C in evaluating the severity and prognosis of patients with severe fever with thrombocytopenia syndrome

**DOI:** 10.1186/s12879-022-07320-7

**Published:** 2022-04-09

**Authors:** Jiao Xie, Shenghua Jie

**Affiliations:** Department of Infectious Diseases, Union Hospital, Tongji Medical College, Huazhong University of Science and Technology, Wuhan, 430022 People’s Republic of China

**Keywords:** Severe fever with thrombocytopenia syndrome, Cystatin C, Prognosis

## Abstract

**Background:**

Severe fever with thrombocytopenia syndrome (SFTS) is a novel emerging viral infectious disease. We explore the value of cystatin C (CysC) level in the evaluation of disease severity and prognosis in patients with SFTS.

**Methods:**

254 patients with SFTS were enrolled in this study. According to the classification and the outcome of the disease, the patients were divided into the general group and the severe group, the severe patients were divided into the fatal group and the non-fatal group. We compared the laboratory indexes by univariate and multivariate logistic regression analysis to explore the severity and prognostic risk factors of SFTS disease, ROC curve and Kaplan–Meier survival analysis curve were drawn to analyze the independent risk factors and the predictive value of disease severity and prognosis.

**Results:**

Univariate analysis showed that the CysC level in severe group and fatal group was significantly higher than general group and non-fatal group (P < 0.05), respectively. Multivariate logistic regression showed that the CysC level was an independent risk factor for severe and death in SFTS patients, and it can effectively predict the risk of severe (AUC = 0.711, 95% CI: 0.645–0.777) and death (AUC = 0.814, 95% CI: 0.737–0.89). The risk of death in patients with cystatin C ≥ 1.23 mg/L was 5.487 times higher than that in patients with cystatin C < 1.23 mg/L.

**Conclusions:**

The CysC level have good predictive value for disease severity and prognosis in patients with SFTS.

*Trial registration* Not applicable

## Background

Severe fever with thrombocytopenia syndrome (SFTS) is an infectious disease caused by neo-Bunia virus infection. It was initially suspected of human granulocytic anaplasmosis. It was not until 2009 that the pathogenic virus was isolated from the blood of a patient in rural areas of Henan Province and was named severe fever with thrombocytopenia syndrome virus (SFTSV) [1]. Until now, cases have been reported in more than 20 provinces in China, as well as in Japan, South Korea and the United States [2]. SFTS is transmitted mainly through tick bites, with an incubation period of about 7–14 days. It has also been reported that SFTS can be transmitted from person to person through contacts with a patient's blood or secretions [3–5].The clinical manifestations of SFTS are not specific, including fever, gastrointestinal symptoms, nervous system symptoms, and decrease of white blood cell (WBC) and platelet (PLT) count. Severe patients can progress to multiple organ dysfunction syndrome (MODS) or even death. The case fatality rate of SFTS is 6.4–20.9% [6, 7]. However, at present, there is no special treatment for SFTSV, such as specific antiviral drugs or vaccines, so it is particularly important to identify the severity of the disease and predict the prognosis of the disease as soon as possible.

Previous studies have shown that viral load, PLT, alanine aminotransferase (ALT), aspartate transaminase (AST), creatine kinase (CK), lactate dehydrogenase (LDH), prothrombin time (PT), activated partial thromboplastin time (APTT), D-dimer (D-D), blood urea nitrogen (BUN) and creatinine (Cr) are related to the prognosis of the disease [8, 9]. It has been confirmed that the level of cystatin C (CysC) is related to the prognosis of many diseases, for example, the increase of CysC level can predict the adverse outcome of patients with COVID-19 and heart failure [10, 11], it also means that stroke recurrence in patients with acute ischemic stroke and the increased risk of rehospitalization in patients with heart failure [11, 12]. It is also a useful indicator for predicting renal prognosis, mortality and acute renal injury in patients with decompensated liver cirrhosis [13, 14], and so on. But there is no research report on the relationship between CysC level and SFTS. Therefore, by collecting the clinical data of patients with SFTS, we explore the value of CysC level in evaluating the severity and prognosis of patients with SFTS, in order to provide some guidance for judging the condition and prognosis of patients with SFTS in clinical work.

## Methods

### Patients

From May 2017 to July 2020, 254 patients with SFTS were collected in the Department of Infectious Diseases, Union Hospital of Tongji Medical College. The enrolled patients were based on the following inclusion criteria: (1) age ≥ 18 years and ≤ 80 years, (2) detection of positive SFTSV ribonucleic acid by reverse-transcriptase polymerase chain reaction (RT-PCR). Patients who met any of the following criteria were excluded: (1) history of chronic kidney disease, (2) history of malignant tumor, (3) autoimmune diseases. According to the clinical manifestations, the patients were divided into two groups: general group and severe group, and the severe group cases were divided into survival group and death group according to the prognosis. The following information were collected and analyzed: demography, laboratory indicators (first blood examination during hospitalization), prognosis. We aimed to explore the value of CysC level in the evaluation of disease severity and prognosis in patients with severe fever with SFTS.

The research was approved by the Ethics Committee of Tongji medical college of Huazhong university of science and technology.

### Statistical analysis

SPSS22.0 and GraphPad Prism 8.0 were used for statistical analysis. Continuous data following normal distribution were expressed the mean ± SD and compared using the t-test; the spacing median and quartile [M (P25 ~ P75)] was used to indicate the non-normal distribution and compared using the Mann–Whitney U test. Categorical data were expressed by rate, and compared using the χ^2^ test. The variables that were significant (P < 0.05) were incorporated into the binary Logistic regression model to obtain independent risk factors and draw the Receiver Operating Characteristic (ROC) curve to evaluate the value of CysC in evaluating the severity of the disease and prognosis. The best cut-off value was calculated according to the ROC curve, and the Kaplan survival Meier survival curve was drawn. Log-rank test was used to calculate the risk ratio (HR) and 95% confidence interval (95% CI). A two-tailed P value less than 0.05 was considered statistically significant.

## Results

### Demographics

A total of 254 patients with SFTS were enrolled in this study. The number of male cases in general group and severe group was 57 (43.8%) and 63 (50.8%) respectively. In the severe group, the mean age was 62.79 ± 9.96 years, which was significantly higher (P = 0.001) than that (median, 58.13 years) of general group patients. Among the severe patients with SFTS, there were 42 (49.4%) males in the survival group, with an average age of 60.71 ± 9.88 years, and 39 patients in the death group, including 21 (53.8%) males, with an average age of 67.30 ± 8.67 years. Significant difference was found in age between the 2 groups (P < 0.001). The details were showed in the Table 1.

**Table 1:** Distribution of SFTS patients between sex and age in different groups

	Total			Severe group		
	General group (N = 130)	Severe group (N = 124)	*P*-value	Non-fatal (N = 85)	Fatal (N = 39)	*P*-value
Sex (male/%)	57 (43.8%)	63 (50.8%)	0.267	42 (49.4%)	21 (53.8%)	0.647
Age (year)	58.13 ± 11.87	62.79 ± 9.96	0.001*	60.71 ± 9.88	67.30 ± 8.67	0.000*

*p < 0.05, the threshold for statistical significance. *SFTS* severe fever with thrombocytopenia syndrome

### Univariate analysis

As showed in the Table 2, in the general group and severe group patients with SFTS, the blood routine indexes are abnormal, which is mainly manifested by the decrease of leukocyte and platelet count. In some cases, the functions of liver, heart, kidney and blood coagulation were impaired, including ALT, AST, ALP, CysC, CK, LDH, D-D and APTT increased in varying degrees. Compared with the general group, it was found that viral load, ALT, AST, BUN, ALP, CysC, D-D,APTT, CK, LDH were higher in the severe group (P < 0.05), while the levels of PLT, Albumin(ALB) and Glomerular filtration rate(GFR) were lower in it (P < 0.05).

**Table 2:** Analysis of laboratory indexes between general group and severe group

		General group (N = 130)	Severe group (N = 124)	*P*-value
SFTSV	(TCID 50/ml)	832 (243–3740)	9655 (1060–83,900)	0.000*
WBC	(G/L)	2.525 (1.71–4.32)	2.745 (1.75–4.35)	0.720
N	(G/L)	1.56 (0.9–2.66)	1.445 (0.77–2.75)	0.479
E	(G/L)	0 (0–0)	0 (0–0)	0.359
L	(G/L)	0.63 (0.4–1.21)	0.695 (0.43–1.02)	0.930
M	(G/L)	0.14 (0.08–0.28)	0.17 (0.07–0.4)	0.956
PLT	(G/L)	57 (42–74)	37 (27–46)	0.000*
ALT	(G/L)	64 (38–104)	108 (62–173)	0.000*
AST	(G/L)	153 (86–232)	321 (177–528)	0.000*
ALP	(U/L)	59.5 (51–80)	67.5 (54–100)	0.003*
ALB	(g/L)	32.65 (29.2–34.7)	29 (26.8–31.8)	0.000*
CysC	(mg/L)	0.945 (0.86–1.08)	1.21 (0.96–1.56)	0.000*
BUN	(mmol/L)	3.84 (2.99–5.4)	5.415 (3.24–7.21)	0.000*
Cr	(μmol/L)	66.8 (58.6–78.4)	67.7 (57.9–90)	0.367
UA	(μmol/L)	219.8 (161.6–275)	219.8 (161.6–275)	0.103
GFR	ml/min/1.71 m^2^	100.0 (87.2–109.8)	95.5 (71.8–104.8)	0.01*
D-D	(mg/L)	2.14 (1.41–3.77)	3.555 (1.97–5.72)	0.000*
PT	(s)	12.7 (12–13.4)	12.85 (12.4–13.9)	0.007*
APTT	(s)	49.3 (45–54.2)	56.95 (48–70)	0.000*
FIB	(g/L)	2.58 (2.28–3.02)	2.59 (2.19–2.99)	0.109
INR		0.97 (0.91–1.04)	0.97 (0.93–1.09)	0.034*
CK	(U/L)	383 (191–814)	914 (419–2114)	0.000*
LDH	(U/L)	623 (427–856)	1258 (763–1697)	0.000*

*p < 0.05, the threshold for statistical significance. *SFTSV* severe fever with thrombocytopenia syndrome virus, *WBC* white blood cell, *N* neutrophil, *E* eosinophil, *L* lymphocyte, *M* monocyte, *PLT* platelet, *ALT* alanine aminotransferase, *AST* aspartate transaminase, *ALB* albumin, *ALP* alkaline phosphatase, *CysC* cystatin C, *BUN* blood urea nitrogen, *Cr* creatinine, *UA* uric acid, *GFR* glomerular filtration rate, *D-D* D-dimer, *PT* prothrombin time, *APTT* activated partial thromboplastin time, *FIB* fibrinogen, *INR* international normalized ratio, *CK* creatine kinase, *LDH* lactate dehydrogenase

Among the severe group patients, they were divided into non-fatal group and fatal group according to their prognosis (death or critical illness gave up treatment). Compared with the non-fatal group, the fatal group showed higher level of viral load, ALT, AST, CysC, Cr, APTT, lower count of Lymphocyte, Mononuclear cell and GFR, and which were statistically different (P < 0.05).The specific data were showed in Table 3.

**Table 3:** Analysis of laboratory indexes between non-fatal group and fatal group

		Non-fatal group (N = 85)	Fatal group (N = 39)	*P*-value
SFTSV	(TCID 50/ml)	3630 (818–14,700)	98,450 (33,750–464,500)	0.000*
WBC	(G/L)	2.89 (1.87–4.35)	2.14 (1.575–4.335)	0.426
N	(G/L)	1.685 (0.75–2.78)	1.365 (0.82–2.645)	0.901
E	(G/L)	0 (0–0)	0 (0–0.01)	0.327
L	(G/L)	0.745 (0.53–1.12)	0.545 (0.32–0.895)	0.019*
M	(G/L)	0.19 (0.08–0.44)	0.085 (0.03–0.21)	0.001*
PLT	(G/L)	38 (29–46)	35 (24–48)	0.222
ALT	(G/L)	102 (54–162)	140.5 (73.5–203.5)	0.039*
AST	(G/L)	277 (160–457)	386 (293–677.5)	0.015*
ALP	(U/L)	68 (51–100)	67 (55.5–103.5)	0.808
ALB	(g/l)	29.15 (27.2–31.8)	28.9 (26.55–31.45)	0.554
CysC	(mg/L)	1.08 (0.87–1.32)	1.6 (1.33–1.825)	0.000*
BUN	(mmol/L)	4.49 (2.8–6.57)	7.165 (6.2–10.03)	0.000*
Cr	(μmol/L)	64.85 (57.9–81.4)	84.6 (62.55–126.7)	0.005*
UA	(μmol/L)	231.9 (156.7–289)	275.95 (209.25–378.65)	0.011*
GFR	ml/min/1.71 m^2^	98.3 (83.4–106.2)	75.1 (50.5–99.3)	0.001*
D-D	(mg/L)	3.555 (1.74–5.42)	3.68 (2.31–9.795)	0.137
PT	(s)	12.7 (12.2–13.5)	13.45 (12.6–14.7)	0.004*
APTT	(s)	54.6 (47.2–65.8)	65.05 (54.5–85.95)	0.000*
FIB	(g/l)	2.62 (2.22–3.1)	2.5 (2.105–2.865)	0.184
INR		0.96 (0.93–1.05)	1.05 (0.95–1.17)	0.020*
CK	(U/L)	937.5 (391–2114)	1029 (634.5–2094)	0.738
LDH	(U/L)	1155.5 (727–1665)	1348 (878.5–1820)	0.227

*p < 0.05, the threshold for statistical significance. *SFTSV* severe fever with thrombocytopenia syndrome virus, *WBC* white blood cell, *N* neutrophil, *E* eosinophil, *L* lymphocyte, *M* monocyte, *PLT* platelet, *ALT* alanine aminotransferase, *AST* aspartate transaminase, *ALB* albumin, *ALP* alkaline phosphatase, *CysC* cystatin C, *BUN* blood urea nitrogen, *Cr* creatinine, *UA* uric acid, *GFR* glomerular filtration rate, *D-D* D-dimer, *PT* prothrombin time, *APTT* activated partial thromboplastin time, *FIB* fibrinogen, *INR* international normalized ratio, *CK* creatine kinase, *LDH* lactate dehydrogenase

In the above analysis, BUN, UA, PT and INR were within the normal range, so the statistical difference was not considered to be of clinical significance.

### Multivariate logistic regression analysis

The variables with significant differences in univariate analysis were further analyzed in the multivariate Logistic regression analysis in Tables 4 and 5. We found that higher CysC was an independent risk factor for the severity of SFTS (P = 0.006), it is also an independent risk factor for death in patients with SFTS (P = 0.003).

**Table 4:** Multivariate Logistic regression analysis between general group and severe group

		B	S.E	Wald	df	Sig	Exp (B)	95% C.I. for EXP (B)	
								Lower	Upper
Age	(Year)	0.005	0.018	0.089	1	0.765	1.005	0.97	1.042
SFTSV	(TCID 50/ml)	0	0	2.742	1	0.098	1	1	1
PLT	(G/L)	− 0.007	0.005	2.471	1	0.116	0.993	0.984	1.002
ALT	(G/L)	− 0.005	0.006	0.643	1	0.423	0.995	0.983	1.007
AST	(G/L)	0.003	0.003	1.412	1	0.235	1.003	0.998	1.009
ALB	(g/L)	− 0.075	0.038	3.93	1	0.057	0.928	0.861	0.999
ALP	(mg/L)	− 0.003	0.007	0.186	1	0.666	0.997	0.984	1.01
CysC	(mg/L)	1.858	0.679	7.49	1	0.006	6.409	1.694	24.244
GFR	ml/min/1.71 m^2^	− 0.001	0.009	0.008*	1	0.927	0.999	0.982	1.016
D-D	(mg/L)	0.026	0.057	0.211	1	0.646	1.027	0.918	1.148
APTT	(s)	0.006	0.018	0.091	1	0.763	1.006	0.97	1.042
CK	(U/L)	0	0	0.367	1	0.545	1	1	1
LDH	(U/L)	0.001	0.001	5.442	1	0.12	1.001	1	1.002
Constant		− 1.609	2.573	0.391	1	0.532	0.2		

*p < 0.05, the threshold for statistical significance. *SFTSV* severe fever with thrombocytopenia syndrome virus, *PLT* platelet, *ALT* alanine aminotransferase, *AST* aspartate transaminase, *ALB* albumin, *ALP* alkaline phosphatase, *CysC* cystatin C, *GFR* glomerular filtration rate, *D-D* D-dimer, *APTT* activated partial thromboplastin time, *CK* creatine kinase, *LDH* lactate dehydrogenase

**Table 5:** Multivariate Logistic regression analysis between non-fatal group and Fatal group

		B	S.E	Wald	df	Sig	Exp (B)	95% C.I. for EXP (B)	
								Lower	Upper
Age	(Year)	0.038	0.032	1.433	1	0.231	1.039	0.976	1.106
SFTSV	(TCID 50/ml)	0	0	0.201	1	0.654	1	1	1
L	(G/L)	− 0.606	0.601	1.019	1	0.313	0.545	0.168	1.77
M	(G/L)	− 1.15	0.963	1.425	1	0.233	0.317	0.048	2.092
ALT	(G/L)	0.005	0.006	0.685	1	0.408	1.005	0.993	1.017
AST	(G/L)	− 0.001	0.002	0.081	1	0.776	0.999	0.996	1.003
CysC	(mg/L)	2.981	1.016	8.608	1	0.003*	19.712	2.69	144.429
Cr	(μmol/L)	0.005	0.009	0.238	1	0.625	1.005	0.986	1.023
GFR	ml/min/1.71 m^2^	0.016	0.012	1.743	1	0.187	1.016	0.992	1.041
APTT	(s)	0.023	0.017	1.909	1	0.167	1.024	0.99	1.058
Constant		− 10.317	2.986	11.941	1	0.001	0		

*p < 0.05, the threshold for statistical significance. *SFTSV* severe fever with thrombocytopenia syndrome virus, *L* lymphocyte, *M* monocyte, *ALT* alanine aminotransferase, *AST* aspartate transaminase, *CysC* cystatin C, *GFR* glomerular filtration rate, *APTT* activated partial thromboplastin time

### ROC curve

**Fig. 1:**
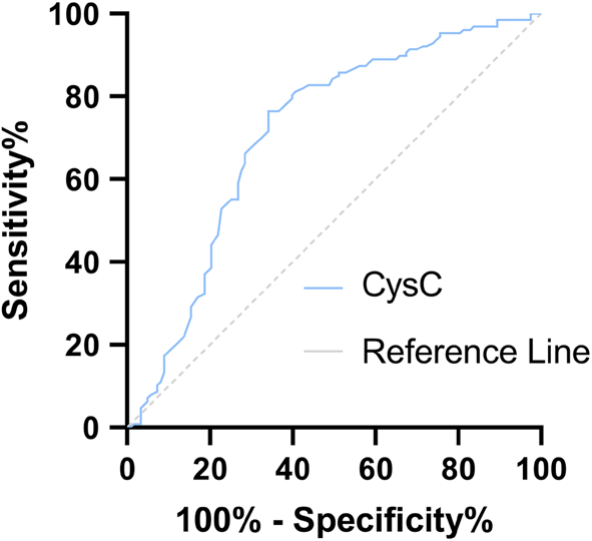
ROC curve of CysC for predicting the risk of severe illness in patients with SFTS

**Fig. 2:**
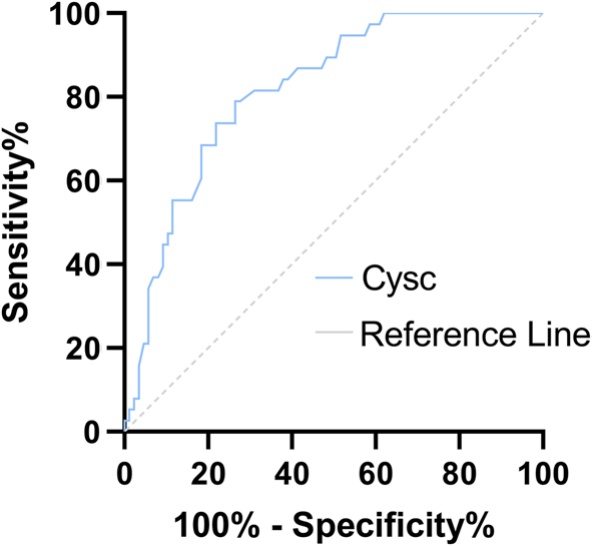
ROC curve of CysC for predicting the risk of death in patients with SFTS

The Area under Curve (AUC) of CysC level to predict severe illness was 0.711 (95% CI: 0.645–0.777, P < 0.05), the sensitivity was 65.9%, and the specificity was 76.4%. The Cut-off value was 1.06 mg/L. The AUC of predicting death was 0.814 (95% CI: 0.737–0.89), the sensitivity was 78.9%, and the specificity was 72.9%. The Cut-off value was 1.23 mg/L (Table 6, Figs. 1, 2).

**Table 6:** The value of cystatin C in predicting severe illness and death in patients with SFTS

	AUC	95% CI	P-value	Cut-off (mg/l)	Sensitivity (%)	Specificity (%)
Severe	0.711	0.645–0.777	< 0.05	1.06	65.9	76.4
Fatal	0.814	0.737–0.89	< 0.05	1.23	78.9	72.9

### Prognostic value of CysC

**Fig. 3:**
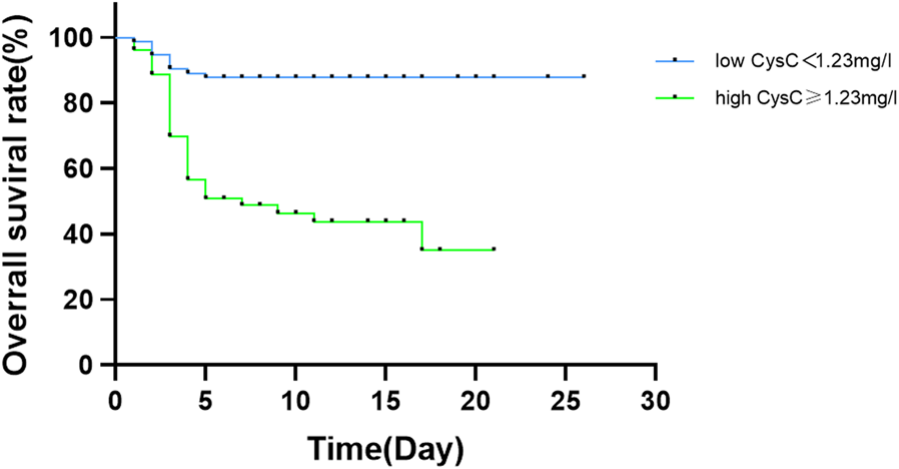
The Kaplan–Meier survival curves of OS between low and high CysC groups

According to the level of CysC, 124 patients with SFTS were divided into two groups: low cystatin C group (N = 71, 57.3%) and high CysC group (N = 53, 42.7%). Compared with the low CysC group (< 1.23 mg/L), the overall survival rate(OS) of high CysC group (≥ 1.23 mg/L) was lower (P < 0.05, HR = 5.487, 95% CI: 2.872–10.48,Fig. 3), and it was statistically significant.

## Discussion

In our study, the clinical data of 254 patients with SFTS were analyzed retrospectively to explore the risk factors affecting the severity and prognosis of the disease. Many previous studies have shown that age is a risk factor for SFTS death [15–17]. Similarly, the age of general group and fatal group in our study was significantly older than that of severe group and non-fatal group, which suggested that the elderly were more likely to develop into severe disease or even death, which may be related to decreased immunity, susceptibility to SFTSV and decreased organ function in the elderly.

SFTSV infection can directly or indirectly damage the function of multiple systems, like most viruses. We found that the viral load, ALT, AST were significantly correlated with the severity of SFTS and the prognosis of critically ill patients, which was consistent with the results of previous studies [18, 19].

To our knowledge, this is the first time to study the relationship between CysC and SFTS. CysC is an endogenous cysteine protease inhibition [20], which is easily detected in biological body fluids and has nothing to do with age, sex, diet, and muscle mass [21]. CysC can regulate the immune response by inhibiting cathepsin activity, reducing MHC-II-mediated antigen presentation [22] and regulating the function of natural killer cells [23]. It can also induce macrophages to release nitric oxide, regulate the degradation of intracellular antigens, and cytokines in T cells and fibroblasts. Further regulate cell differentiation, proliferation and biological activity [24–26]. It was found that the level of CysC was positively correlated with microinflammatory indexes such as interleukin (IL)-1β, IL-6, tumor necrosis factor (TNF)-α and C reactive protein (CRP) It was also suggested that the elevated level of CysC activated mononuclear macrophage system, endothelial cells or neutrophils to some extent, resulting in excessive release of inflammatory cytokines (such as IL-6, TNF, etc.), thus promoting the occurrence of inflammatory reaction [27]. Therefore, high levels of CysC may reflect high levels of inflammation and immune response in the body.

We found that the level of CysC was significantly correlated with the severity and prognosis of SFTS. The risk of death in high CysC group (≥ 1.23 mg/L) was significantly higher than that in low CysC group (< 1.23 mg/L) (HR = 5.487), and other renal function indexes were not independent risk factors for disease severity and risk of death. At the same time, previous studies have found the association between septicemia and high serum CysC levels, and the association trend between Acute Physiology and Chronic Health Evaluation II(APACHE II) and cystatin C [28], which may represent direct and indirect inflammation, as some authors have suggested, CysC may reflect the pathogenic state other than GFR [29]. Therefore, CysC, as an index that is easy to detect and relatively unaffected, can provide a certain value in judging the severity and prognosis of patients with SFTS, which undoubtedly provides a more convenient basis for clinical work to judge the severity of the disease and prognosis. However, the role and regulatory mechanism of CysC in SFTS need to be further studied and verified.

However, this study also has some limitations. First, this study is a single-center retrospective study. Some patients were not included in the study due to the lack of data, which means there is a certain selection bias. Secondly, there is no dynamic monitoring of laboratory indicators to better judge the relationship between index changes and diseases. Finally, the collection of cases is limited, and the representativeness of the results needs to be further expanded to verify the sample.

## Conclusions

The level of cystatin C was independently correlated with the severity of SFTS disease and the risk of death in hospital. The risk of death in critically ill patients with cystatin C level ≥ 1.23 mg/L was 5.487 times higher than that in patients with cystatin level lower than 1.23 mg/L. Therefore, we believe that the cystatin C level plays an important role in evaluating the severity and prognosis of patients with SFTS, but the role and regulatory mechanism of cystatin C in SFTS need to be further studied and verified.

## Data Availability

The datasets used in the study are available from the corresponding author on reasonable request.

## Abbreviations

SFTS: Severe fever with thrombocytopenia syndrome
SFTSV: Severe fever with thrombocytopenia syndrome virus
WBC: White blood cell
PLT: Platelet
MODS: Multiple organ dysfunction syndrome
ALT: Alanine aminotransferase
AST: Aspartate transaminase
CK: Creatine kinase
LDH: Lactate dehydrogenase
PT: Prothrombin time
APTT: Activated partial thromboplastin time
D-D: D-dimer
BUN: Blood urea nitrogen
Cr: Creatinine
CysC: Cystatin C
RT-PCR: Reverse-transcriptase polymerase chain reaction
ROC: Receiver operating characteristic
HR: Risk ratio
95% CI: 95% Confidence interval
N: Neutrophil
E: Eosinophil
L: Lymphocyte
M: Monocyte
ALP: Alkaline phosphatase
ALB: Albumin
GFR: Glomerular filtration rate
UA: Uric acid
INR: International normalized ratio
AUC: Area under curve
DIC: Disseminated intravascular coagulation
OS: Overall survival
IL: Interleukin
TNF: Tumor necrosis factor
CRP: C reactive protein
APACHE II: Acute Physiology and Chronic Health Evaluation II
